# Poor Cervical Cancer Knowledge and Awareness among Women and Men in the Eastern Cape Province Rural Community

**DOI:** 10.3390/ijerph20206916

**Published:** 2023-10-13

**Authors:** Zizipho Z. A. Mbulawa, Lindelo L. Mahlangu, Esihle Makhabane, Sisanda Mavivane, Sindisiwe Nongcula, Anathi Phafa, Ayabonga Sihlobo, Mbalentle Zide, Athenkosi Mkiva, Thembeka N. Ngobe, Luxolo Njenge, Phumla Kwake, Charles B. Businge

**Affiliations:** 1 National Health Laboratory Service, Nelson Mandela Academic Hospital, Mthatha 5100, South Africa; 2 Department of Laboratory Medicine and Pathology, Faculty of Health Sciences, Walter Sisulu University, Mthatha 5100, South Africa; 3 UCT-SAMRC Gynaecological Cancer Research Centre, University of Cape Town, Cape Town 7700, South Africa; cbusinge@wsu.ac.za; 4 MBChB-3 2022 COBES Group, Faculty of Health Sciences, Walter Sisulu University, Mthatha 5100, South Africa; 220133131@mywsu.ac.za (L.L.M.); 220114188@mywsu.ac.za (E.M.); 220108684@mywsu.ac.za (S.M.); 220116091@mywsu.ac.za (S.N.); 220099200@mywsu.ac.za (A.P.); 220115672@mywsu.ac.za (A.S.); 219053243@mywsu.ac.za (A.M.); 220295352@mywsu.ac.za (T.N.N.); 220099898@mywsu.ac.za (L.N.); 5 Tabase Community Health Centre, Eastern Cape Department of Health, Mthatha 5108, South Africa; pumla.kwake@gmail.com; 6 Department of Obstetrics and Gynaecology, Nelson Mandela Academic Hospital, Mthatha 5100, South Africa; 7 Department of Obstetrics and Gynaecology, Faculty of Health Sciences, Walter Sisulu University, Mthatha 5100, South Africa

**Keywords:** cervical cancer, knowledge, men, women, cervical cancer screening

## Abstract

Cervical cancer knowledge and awareness is low among South Africans despite high cervical cancer prevalence. This study aimed to investigate knowledge about the symptoms, signs, risk factors, and methods of prevention of cervical cancer among women and men in the rural Eastern Cape province, South Africa. This cross-sectional analytical study was conducted in the rural community of the OR Tambo municipality in the Eastern Cape province. 252 women and men aged ≥ 25 years were randomly recruited. Data were collected using semi-structured questionnaires. A knowledge score was categorized as “good” if it was ≥65%. The majority of participants (69.8%) were women. Only a proportion of 25.6% (51/199) of the participants had good overall knowledge about cervical cancer, and the majority of these (84.3%) were women. Women previously screened for cervical cancer had a significantly higher median cervical knowledge score than those who had never been screened (*p* = 0.002). Only among women, good knowledge about cervical cancer was associated with a tertiary education level (OR: 3.17, 95% CI: 1.08–9.57, *p* = 0.044) and high household income (OR: 3.40, 95% CI: 1.24–9.75, *p* = 0.027). Both women and men in rural Eastern Cape had limited knowledge about the risk factors and prevention methods of cervical cancer. Public health strategies to improve knowledge and awareness of cervical cancer among both men and women are necessary.

## 1. Introduction

Cervical cancer is the second most common cancer among South African women; furthermore, in 2020, it was reported to be the most common cause of cancer mortality in this population [1,2]. The incidence of cervical cancer is reported to be increasing in the rural Eastern Cape province of South Africa [3]. More than 63% of new cases of cervical cancer occur among women living with human immunodeficiency virus (HIV) [4,5]. South Africa is a country with high HIV prevalence, with a 24.5 (95% CI: 20.8–27.7) prevalence rate among women aged 15–49 years in 2021 [6].

Cervical cancer is among the cancers that can be prevented by effective screening, early diagnosis, and the treatment of cervical precancerous lesions [7,8,9,10]. Low uptake of cervical cancer screening is associated with missed opportunities for the treatment of high-grade cervical precancerous lesions and initial diagnosis of cervical cancer at an early stage [11]. The South African Cervical Cancer Prevention and Control Policy states that women should receive free cervical cytology screening starting at 25 years of age using liquid-based cytology at five-year intervals. It further stipulates that high-risk women, such as HIV-positive women, should be screened upon HIV diagnosis and at three-year intervals thereafter [1]. HR-HPV molecular testing, though widely available in private practice, however, has not yet been incorporated as one of the cervical cancer screening methods in the public sector.

Although self-sampling screening methods have been reported to show the potential to increase cervical cancer screening coverage [12], these have also not yet been incorporated into the South African national cervical cancer screening program [11,13]. Despite studies that have shown that South African women have a positive attitude towards self-sampling, clinicians collecting cervical specimens is the main method currently being used [10]. There are currently no active call and recall systems in South Africa for cervical cancer screening; furthermore, loss of follow-up before treatment is reported to be high [10].

Among the general South African population, limited awareness and knowledge about cervical cancer, its risk factors, and the available prevention measures contribute to low screening coverage and high cervical cancer burden [13,14,15,16,17,18,19,20,21,22]. This is not only limited to the South African population but seems to be the norm in other sub-Saharan countries where knowledge about cervical cancer has similarly been reported to be poor [23,24]. It is common for men to have poor knowledge of and misperceptions about cervical cancer prevention and other women’s health issues; yet, men in sub-Saharan Africa control the economic resources and make most health-related decisions for their families and population [25,26,27,28]. South African primary healthcare workers have been reported to have inadequate knowledge about cervical cancer prevention and treatment services, which may result in them giving low-quality health education to individual patients and communities around public health facilities [14,15,16].

Following the approval of HPV vaccines in South Africa in 2008, a high vaccine coverage rate was reported [9,29]. Despite this high HPV vaccination coverage, cervical cancer screening is still necessary to eliminate cervical cancer, especially among the population of women who are above the prescribed age for HPV vaccination [7,30]. Considering the high cervical cancer and HIV burden in South Africa, and the high incidence of cervical cancer attributable to HIV, the primary prevention methods such as HIV and cervical cancer prevention, health education, and screening are important and their enhancement is still required. A systematic review by Makadzange et al. reported that among African women, cervical cancer prevention educational interventions increased knowledge and awareness and increased cervical cancer screening uptake [19]. Therefore, this study aimed to investigate the level of knowledge of the symptoms, signs, risk factors, and the methods of prevention of cervical cancer among women and men residing in the rural Eastern Cape province, South Africa.

## 2. Materials and Methods

### 2.1. Ethical Statement

Participation in the study was voluntary and written informed consent was obtained from all study participants before data collection. This study was conducted in accordance with the Declaration of Helsinki, and approved by the Human Research Ethics and Biosafety Committee of Walter Sisulu University (reference: 088/2022).

### 2.2. Study Setting, Population, and Recruitment

This was a cross-sectional study conducted among women and men of the rural community located in the OR Tambo municipality, Eastern Cape, South Africa. The study was conducted at the public healthcare facility, between October and November 2022. Women and men aged 25 years and above visiting the public health facility for any reason were recruited to participate in the study. Participants were recruited from the reception through the use of posters and brochures.

### 2.3. Data Collection

This study was a component of Walter Sisulu University’s Community-based Education and Service (COBES) program, aiming to expose MBChB third-year students to community-specific health issues and introduce them to health-related research. Ten Walter Sisulu University Bachelor of Medicine and Bachelor of Surgery (MBChB) third-year-level students were trained to recruit study participants and administer the study questionnaires. To collect information on knowledge and awareness of cervical cancer, its risk factors, and prevention methods, a face-to-face interview was conducted and questionnaires were administered by the investigators. The questionnaires consisted of four sections: the first section focused on socio-demographic questions, the second section focused on knowledge and awareness of cervical cancer, the third section focused on cervical cancer risk factors, and the fourth section focused on cervical cancer prevention methods. The questionnaires were designed based on published studies [17,18,28] and peer-reviewed.

### 2.4. Data Analysis

All variables were captured and coded in Microsoft Excel 2016. Categorical variables were presented using frequency tables, percentages, and graphs. Numerical data that were not normally distributed were reported using the median and interquartile range (IQR). A knowledge score was categorized as “good” if it was greater than or equal to 65% or as “poor” when the knowledge score was less than 65%. The cut-off was based on previous cervical cancer knowledge and awareness studies in the Eastern Cape province of South Africa [14,15]. The knowledge level and score analysis included only participants who reported having heard about cervical cancer. A Mann–Whitney test was used to compare the knowledge scores between groups. A chi-square test was used to analyze categorical data. Binary logistic regression was used to investigate the associations between knowledge and sociodemographic characteristics. The results were reported as odds ratios (OR) and 95% confidence intervals (95% CI). The level of significance was set at 5% (*p*-value ≤ 0.05).

## 3. Results

### 3.1. Characteristics of Study Participants

A total of 252 women and men participated in the study. All participants belonged to the black ethnicity group, whose home language is IsiXhosa. The majority of study participants were women (69.8%, 176/252). The women’s median age was 37 years with an interquartile range (IQR) of 30–49 years, while the median age for men was 39 years (IQR: 30–50 years). Ages were not found to differ between the women and men (*p* = 0.287). Women had a higher, but non-significant, proportion of higher education than men (25.6% and 18.4%, respectively, *p* = 0.258). A higher proportion of men were employed compared to women (52.6% and 31.3%, respectively, *p* = 0.002). Most participants (46.0%) reported having <R1999 monthly household income and more women than men were in this group (*p* = 0.039). A proportion of 39.2% reported having participated in cervical cancer screening at least once in their lifetime (Table 1).

### 3.2. Knowledge about Cervical Cancer and Its Risk Factors According to Gender

A high percentage of participants (79.0%) reported having heard of cervical cancer; more women (83.5%) reported having heard about cervical cancer than men (68.4%, *p* = 0.011). Participants reported having heard of cervical cancer were more likely to have a tertiary education level (*p* < 0.001) and be younger (*p* = 0.005) than those who had not heard about cervical cancer. The sources of cervical cancer information were reported to be hospital staff (47.2%), radio/television/internet (30.2%), and friends/relatives (24.6%). A significantly higher proportion of women reported having heard about cervical cancer from hospital staff than men (54.4% and 26.9%, respectively, *p* = 0.001). Among men, a higher proportion reported having heard about cervical cancer from the radio/television/internet than women (44.2% and 25.2%, respectively, *p* = 0.014).

Among participants who had heard of cervical cancer, almost half of the participants (53.8%) reported that cervical cancer is very common and only 43.2% of participants reported that cervical cancer is caused by HPV. A proportion of 42.7% reported that HPV infection is common. Approximately half of the participants stated that HPV infection increases the risk of cervical cancer development and this knowledge was more common among women than men (50.3% and 34.6%, respectively, *p* = 0.032). Smoking and multiple sexual partners were reported to be the risk factors of cervical cancer (53.3% and 77.9%, respectively) (Table 2). The majority of women reported that they were not at risk of cervical cancer (81.9%) and this was not found to be influenced by age.

### 3.3. Knowledge about Cervical Cancer Prevention Methods According to Gender

**Figure 1:**
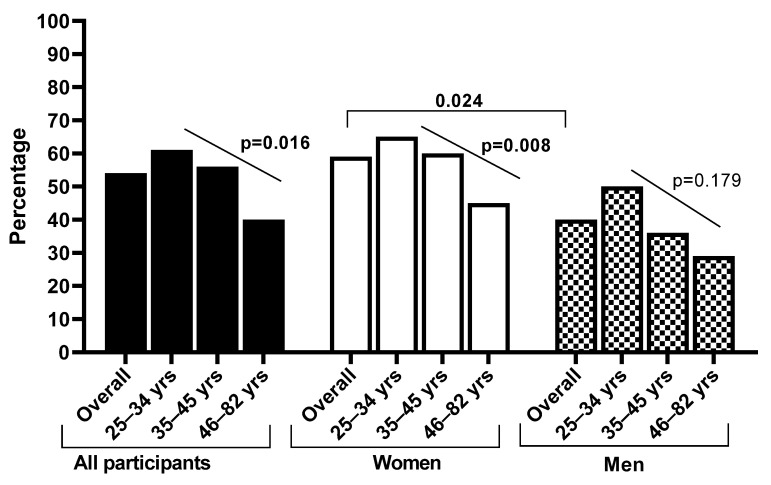
Knowledge about Pap smear as a cervical cancer screening test among Eastern Cape rural community women and men who had heard of cervical cancer.

Among participants who had heard of cervical cancer, 53.8% reported that a Papanicolaou (Pap) smear is used to screen for cervical cancer, and this knowledge was more common among women (58.5%) than men (40.4%, *p* = 0.024). This knowledge decreased with increasing age among all participants combined (*p* = 0.016) and among women (*p* = 0.008) but not among men alone (*p* = 0.179, Figure 1). The younger age group (25–34 years) among the men was more likely to report that Pap smear is used to screen for cervical cancer than other age groups. A proportion of 79.9% participants stated that cervical cancer screening can detect cervical lesions. HPV vaccination was reported to be one of the methods that can be used for cervical cancer prevention by 46.7% of the participants. Approximately a third of the participants stated that cervical cancer cannot be prevented (34.2%) and 61.3% were aware that cervical cancer treatment is available (Table 3).

### 3.4. Cervical Cancer Knowledge among Women According to Cervical Cancer Screening Status

Table 4 presents selected knowledge of cervical cancer, its risk factors, and prevention methods between screened and unscreened women who had heard about cervical cancer. The women previously screened for cervical cancer compared to women who had never been screened were more likely to report that HPV causes cervical cancer (*p* = 0.003), HPV infection increases the risk of cervical cancer (*p* = 0.005), and Pap smear is used for cervical cancer screening (*p* < 0.001). Meanwhile, women not previously screened for cervical cancer were more likely to report that early sexual debut increases the risk of cervical cancer (*p* = 0.023, Table 4).

### 3.5. Cervical Cancer Knowledge Score among Women and Men

Women and men who reported not having heard of cervical cancer (16.5% and 31.6%, respectively) were not included in the cervical cancer knowledge score analysis. A proportion of 25.6% (51/199) had a good knowledge score of cervical cancer, its risk factors, and prevention methods; the majority (84.3%) were women, and men made up 15.7% (*p* < 0.001). The median knowledge score was higher among women than men but not statistically significant (median: 52.0, IQR: 39.0–65.0; median: 48.0, IQR: 35.0–60.0, respectively, *p* = 0.181).

**Figure 2:**
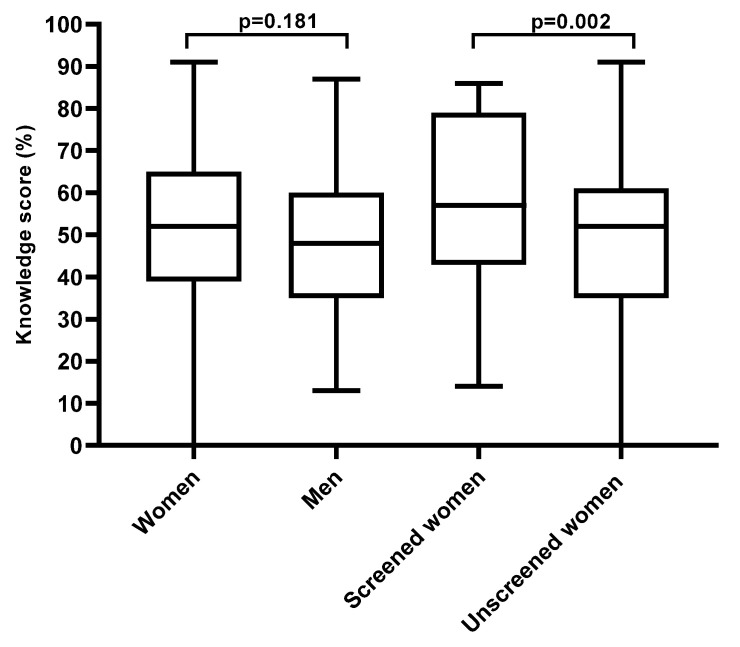
Knowledge score of cervical cancer, its risk factors, and prevention methods among Eastern Cape rural community women and men who had heard of cervical cancer.

Women were further stratified according to ever having been screened for cervical cancer. Only 35.3% of the women previously screened for cervical cancer had a good cervical cancer knowledge score (≥65). This was not significantly higher than that of the women who had never been screened for cervical cancer (24.1%, *p* = 0.149). However, the median cervical cancer knowledge score of the previously screened women was significantly higher than that of the women who had never been screened for cervical cancer (median: 57.0, IQR: 43.0–79.0; median: 52.0, IQR: 35.0–61.0, respectively, *p* = 0.002, Figure 2).

The knowledge score among participants aged 46–82 years was significantly lower than participants aged 25–34 years (OR: 0.36, 95% CI: 0.16–0.85, *p* = 0.019). The participants with tertiary education were more likely to have a good knowledge score than those with primary education (OR: 2.86, 95% CI: 1.03–7.18, *p* = 0.039); a similar trend was observed among women when participants were stratified according to gender (OR: 3.40, 95% CI: 1.24–9.75, *p* = 0.027). Among women, a good cervical knowledge score was associated with a high household income (>R10000, which is approximately >$528) more than a low household income (<R1999, which is approximately <$105; OR: 3.17, 95% CI: 1.08–9.57, *p* = 0.044). Among men, age, education level, employment status, and household income did not impact a good cervical knowledge score.

## 4. Discussion

Overall, this study finds that both women and men in rural Eastern Cape, South Africa had limited knowledge and awareness about cervical cancer, its risk factors, and prevention methods even though they reported having heard of cervical cancer. Similar findings have been reported elsewhere [13,14,15,16,17,18,19,20,21,22,23,24,26,27,31,32]. It was apparent that the level of knowledge about cervical cancer decreased with increasing age among rural community women and men. This could be because the young generation is more informed about health issues in school and media than the older generation [33]. Among women, tertiary education level and high household income were associated with good knowledge, and similar findings have also been reported elsewhere [17,34]. Women previously screened for cervical cancer demonstrated better knowledge than unscreened women about the risk factors and prevention methods of cervical cancer. This can be attributed to the regular health education provided in health facilities during cervical cancer screening. These findings were consistent with those reported elsewhere [17]. It has been reported that among African women, cervical cancer prevention educational interventions increased knowledge, awareness, and cervical cancer screening uptake [21].

Improving knowledge about cervical cancer among men can contribute to cervical cancer prevention since most women will need their husband’s or fathers’ social and economic support, including approval to attend health services. Men with adequate cervical cancer knowledge could encourage women to have regular cervical cancer screening, which would improve the probability of appropriate diagnosis and treatment of high-grade premalignant cervical lesions and early diagnosis and treatment of cervical cancer when it was still in early curable stages [26]. Intimate partner emotional support has been associated with cervical cancer screening uptake [35]. Mutyaba et al. reported that involving male partners resulted in reduced loss of follow-up after cervical cancer screening [36].

The women and men who participated in this study represent those in the larger community in the rural Eastern Cape province from whom consent is often sought before girls aged 9–14 years old are vaccinated during the national HPV school-based vaccination program. Limited knowledge and awareness about cervical cancer in general, and HPV prevention methods in particular, may hamper them from providing consent for their daughters to receive HPV vaccination. This can potentially preclude high HPV vaccine coverage in the study area since the school-based South African national HPV vaccination program uses a school-class-based strategy with an opt-out consent form process [9,10,29]. In South Africa, this is a real threat to successful HPV vaccination as the school-based HPV vaccination program relies on parents’/guardians’ consent. Amponsah-Dacosta and colleagues reported a downward trend in vaccine coverage and dose completion rates since the South African school-based HPV vaccination program’s inception in 2014 [29]. These authors thought that limited knowledge about cervical cancer among parents/guardians could have led to a high rate of non-consent for their children to be vaccinated against HPV, in addition to other factors [29].

Approximately three-quarters of the women participating in the study did not consider themselves at risk of the development of cervical cancer. Because this population perceive themselves as not at risk, they are more likely not to participate in cervical cancer screening and miss the opportunity for early disease detection and treatment [31]. It is worrisome that the women who did not consider themselves at risk of cervical cancer had a lower knowledge score about cervical cancer and its risk factors than those who considered themselves at risk of cervical cancer development. Our results are similar to those previously reported, which are that the level of cervical cancer knowledge is reported to be linked to the way women perceive themselves as at risk of cervical cancer development [31,32]. Our findings agree with earlier observations made by other authors that personal barriers, in addition to healthcare system and sociocultural barriers, affect cervical cancer prevention and treatment programs [26,32]. Low cervical cancer screening uptake limits the chances of picking up cervical precancerous lesions early and initiating treatment at an early stage. Due to this, women seek medical care when cervical cancer is at an advanced stage and the prognosis is poor [9]. Despite free cervical cancer screening services, in 2020, cervical cancer was the most common cause of cancer mortality in South Africa [2]. Misconception about cervical cancer prevention methods was also noted among women and men of rural Eastern Cape. Furthermore, approximately a third of the study population reported that the removal of the uterus, cervix, or upper vagina as part of cervical cancer treatment would make women incomplete. This misperception can have a negative effect on the uptake of cervical cancer screening and treatment [37].

### 4.1. Strength of the Study

According to our knowledge, this was the first study to concurrently investigate the knowledge and awareness about the risk factors, prevention, and treatment of cervical cancer among women and men aged ≥ 25 years from the same rural community in the Eastern Cape province. Understanding the views and attitudes of men on cervical cancer could be useful for designing successful public interventions for cervical cancer prevention. The participants’ home language was used to minimize the possible bias that could be introduced by language barriers.

### 4.2. Limitations of the Study

Only 30.2% of the participants were men. During the study period, the community health facility from which participants were recruited was highly attended by women, and recruiting the available men was more challenging than recruiting women. It was more likely for men to not agree to participate in the study than women. This could be because cervical cancer is viewed as a disease that affects women only. The total number of women and men invited was not noted; therefore, it was not possible to calculate the total response rate. The results observed in this study could be affected by the limited sample size, especially of the male population. Since the project was associated with the MBChB-3 COBES yearly program, the time frame for recruiting study participants and collecting the data was limited. Increased allocated time could have resulted in the mobilization of men to participate in the study. Due to the nature of the study, some of the participants may have been embarrassed to provide accurate answers.

### 4.3. Expected Outcomes and Impact of the Study

This study is the first to report on the knowledge and awareness of the risk factors and methods of prevention of cervical cancer among women and men (≥25 years) residing in the rural community in the Eastern Cape province, South Africa. The data generated from this study report the high level of inaccurate information among the general population’s women and men, which could contribute to low cervical cancer screening uptake. Furthermore, this decreases chances of high-grade premalignant cervical lesions and cervical cancer being diagnosed early. These study findings will inform the South African Department of Health’s policymakers and contribute to the design and implementation of cervical cancer health promotion strategies, which will benefit both women and men in future. Cervical cancer health promotion strategies must be directed to all genders and benefit cervical cancer prevention (screening and HPV vaccination) and treatment methods.

## 5. Conclusions

Both women and men in rural Eastern Cape seem to have limited knowledge about the risk factors and prevention methods of cervical cancer. Women previously screened for cervical cancer demonstrated better knowledge and awareness about cervical cancer than unscreened women. Good knowledge was associated with a tertiary education level and high household income among women but not in men. Public health strategies to improve knowledge and awareness of cervical cancer among both men and women would be more likely to contribute to the uptake of HPV vaccination, cervical cancer screening, and early diagnosis and management of cervical intraepithelial lesions, as well as early-stage cervical cancer.

## Figures and Tables

**Table 1:** Characteristics of Eastern Cape rural community study participants on knowledge, and awareness of cervical cancer and its risk factors, prevention, and treatment methods.

		All Participants, N = 252		Women, N = 176		Men, N = 76		
Variable		%	n	%	n	%	n	*p*-Value
Age	25–34 years	40.5	102	40.3	71	40.8	31	>0.999
	35–45 years	28.6	72	30.1	53	25.0	19	0.450
	46–82 years	31.0	78	29.5	52	34.2	26	0.462
Education level	Primary (Grade 0–7)	26.2	66	25.6	45	27.6	21	0.756
	Secondary (Grade 8–12)	50.4	127	48.9	86	53.9	41	0.494
	Tertiary	23.4	59	25.6	45	18.4	14	0.258
Employment status	Employed	37.7	95	31.3	55	52.6	40	**0.002**
	Unemployed	59.9	151	66.5	117	44.7	34	**0.002**
	Student	2.4	6	2.3	4	2.6	2	>0.999
Household income	<R1999	46.0	116	50.6	89	35.5	27	**0.039**
	R2000–R5000	29.0	73	29.5	52	27.6	21	0.880
	R5001–R10000	8.7	22	6.3	11	14.5	11	0.050
	>R10001	12.7	32	3.4	6	34.2	26	**<0.001**
	Missing data	3.6	9	1.1	2	9.2	7	**0.004**
Done Pap smear? (only females)	Yes			39.2	69			
	No			60.2	106			
	Missing data			0.6	1			

The *p*-values in bold are significant < 0.05.

**Table 2:** Knowledge about cervical cancer and its risk factors among women and men who had heard of cervical cancer in the Eastern Cape rural community.

		Overall		Women		Men		
Variable		%	n	%	n	%	n	*p*-Value *
How common is cervical cancer?	Very common to common *	53.8	107	52.4	77	57.7	30	0.509
	Very rare to rare	26.6	53	29.3	43	19.2	10	0.160
	Do not know	19.6	39	18.4	27	23.1	12	0.462
What causes cervical cancer?	HPV *	43.2	86	43.4	66	38.5	20	0.421
	HIV	13.1	26	13.8	21	9.6	5	0.479
	TB	3.0	6	2.6	4	3.8	2	0.381
	Bacteria	13.1	26	11.8	18	15.4	8	0.564
	Do not know	31.7	63	30.3	46	32.7	17	0.852
Early stages of cervical cancer do not have signs/symptoms	Yes	26.6	53	27.9	41	23.1	12	0.500
	No *	20.1	40	20.4	30	19.2	10	0.856
	Do not know	53.3	106	51.7	76	57.7	30	0.246
Signs/symptoms of cervical cancer	Pain during urination *	43.2	86	49.0	72	26.9	14	**0.006**
	Excessive vaginal bleeding after sex *	32.7	65	36.1	53	23.1	12	0.086
	Vaginal itchiness	20.1	52	32.0	47	9.6	5	**0.007**
	Normal vaginal discharge	13.1	26	17.0	25	1.9	1	**0.006**
	Missing menstruation	9.5	19	10.2	15	7.7	4	0.596
	Don’t know	39.2	78	32.0	47	40.4	21	0.272
Which of the following increases the risk of cervical cancer?	HPV infection *	46.2	92	50.3	74	34.6	18	**0.032**
	Tuberculosis	7.5	15	6.8	10	9.6	5	0.509
	Frequent sex with one man	13.1	26	13.6	20	11.5	6	0.704
	Giving birth to one child	3.5	7	4.8	7	0.0	0	…
	Smoking *	24.6	49	25.9	38	21.2	11	0.500
	Family history of CC *	10.1	20	10.9	16	7.7	4	0.511
	HIV and other STIs *	31.7	63	33.3	49	26.9	14	0.393
	Early sexual activity *	24.1	48	25.2	37	21.2	11	0.561
How common is HPV infection?	Very common *	42.7	85	46.3	68	32.7	17	0.089
	Very rare	26.6	53	25.9	38	28.8	15	0.675
	Do not know	30.7	61	27.9	41	38.5	20	0.155
HPV is transmitted by…	Sexual intercourse *	61.8	123	65.3	96	51.9	27	0.089
	Skin-to-skin contact *	1.5	3	0.7	1	3.8	2	0.168
	Droplets or sneezing	2.0	4	2.7	4	0.0	0	…
	Contaminated surfaces	5.0	10	5.4	8	3.8	2	>0.999
	By any forms	0.5	1	0.7	1	0.0	0	…
	Do not know	29.6	59	25.9	38	40.4	21	0.049
Is smoking a risk factor for cervical cancer?	Yes *	53.3	106	52.4	77	55.8	29	0.674
	No	11.6	23	14.3	21	3.8	2	**0.043**
	Do not know	35.2	70	33.3	49	40.4	21	0.360
Is having multiple sexual partners a risk factor for cervical cancer?	Yes *	77.9	155	74.8	110	86.5	45	0.080
	No	3.0	6	4.1	6	0.0	0	…
	Do not know	19.1	38	21.1	31	13.5	7	0.229

CC: Cervical cancer. * Indicates the correct response; some questions have multiple correct responses. The *p*-values in bold are significant < 0.05.

**Table 3:** Knowledge about cervical cancer prevention methods among Eastern Cape rural community women and men who had heard of cervical cancer.

		Overall		Women		Men		
Variable		%	n	%	n	%	n	*p*-Value *
What test is used for cervical cancer screening?	Pap smear *	53.8	107	58.5	86	40.4	21	**0.024**
	Urine test	4.5	9	4.8	7	3.8	2	>0.999
	X-ray	6.5	13	6.8	10	5.8	3	>0.999
	There is no test to screen for CC	1.0	2	1.4	2	0.0	0	…
	Do not know	20.1	40	15.0	22	34.6	18	**0.002**
How can cervical cancer be prevented?	HPV vaccination *	46.7	93	49.0	72	40.4	21	0.286
	Abstinence	16.1	32	17.0	25	13.5	7	0.550
	Healthy diet	10.6	21	10.7	16	9.6	5	0.798
	Exercise	1.5	3	1.4	2	1.9	1	>0.999
	Screening using Pap smear *	13.1	26	17.0	25	1.9	1	**0.004**
	Don’t know	24.6	49	21.1	31	34.6	18	0.052
Cervical cancer cannot be prevented	True	34.2	68	33.3	49	36.5	19	0.675
	False *	64.3	128	64.6	95	63.5	33	0.880
	Don’t know	1.5	3	2.0	3	0.0	0	…
Screening can detect cervical lesions so they do not develop into cancer	True *	79.9	159	81.0	119	76.9	40	0.533
	False	191	38	17.7	26	23.1	12	0.395
	Don’t know	1.0	2	1.4	2	0.0	0	…
HPV vaccine can prevent cervical cancer	True *	76.4	152	76.2	112	76.9	40	0.915
	False	21.6	43	21.1	31	23.1	12	0.847
	Don’t know	2.0	4	2.7	4	0.0	0	…
The use of condoms can help prevent HPV infection	True *	85.4	170	85.7	126	84.6	44	0.847
	False	13.1	26	12.2	18	15.4	8	0.564
	Don’t know	1.5	3	2.0	3	0.0	0	…
Is there a treatment for cervical cancer?	Yes *	61.3	122	63.3	93	55.8	29	0.340
	No	11.6	23	10.2	15	9.6	5	0.903
	Maybe	10.6	21	8.8	13	15.4	8	0.187
	Do not know	16.6	33	17.7	26	13.5	7	0.481

CC: Cervical cancer. * Indicates the correct response; some questions have multiple correct responses. The *p*-values in bold are significant < 0.05.

**Table 4:** Knowledge about cervical cancer among screened and unscreened women who had heard of cervical cancer in the Eastern Cape rural community.

	Screened Women, N = 68		Unscreened Women, N = 79		
	%	n	%	n	*p*-Value
HPV causes cervical cancer.	58.8	40	32.9	26	**0.003**
Early stages of cervical cancer do not have signs/symptoms.	35.3	24	21.5	17	0.068
HPV infection increases the risk of cervical cancer.	63.2	43	39.2	31	**0.005**
Smoking increases the risk of cervical cancer.	22.1	15	27.8	22	0.451
HIV and other STIs increases cervical cancer risk.	35.3	24	32.9	26	0.862
Early sexual debut increases the risk of cervical cancer.	16.2	11	32.9	26	**0.023**
HPV infection is common.	51.5	35	41.8	33	0.251
HPV is sexually transmitted.	72.1	49	59.5	47	0.121
Having multiple sexual partners a risk factor for cervical cancer.	79.4	54	70.9	56	0.258
Pap smear is used for cervical cancer screening.	79.4	54	40.5	32	**<0.001**
Cervical cancer can be prevented by screening using a Pap smear.	16.2	11	12.7	10	0.639
Cervical cancer can be prevented by HPV vaccination.	55.9	38	44.3	35	0.187
Cervical cancer can be prevented.	72.1	49	58.2	46	0.087
Screening can detect cervical lesions so they do not develop into cancer.	83.8	57	78.5	62	0.528
The HPV vaccine can prevent cervical cancer.	77.9	53	74.7	59	0.700
The use of condoms can help prevent HPV infection.	89.7	61	82.3	65	0.242
There is treatment for cervical cancer.	60.3	41	65.8	52	0.498

The *p*-values in bold are significant < 0.05.

## Data Availability

Data are available on request from the principal investigator.
